# Childhood Traumatic Experiences and Dimensional Models of Personality Disorder in DSM-5 and ICD-11: Opportunities and Challenges

**DOI:** 10.1007/s11920-021-01265-5

**Published:** 2021-07-19

**Authors:** Sarah N. Back, Aleya Flechsenhar, Katja Bertsch, Max Zettl

**Affiliations:** 1 Department of Psychology, Faculty of Psychology and Education, Ludwig-Maximilians-University Munich, Leopoldstraße 13, 80802 Munich, Germany; 2 Department of General Psychiatry, Center for Psychosocial Medicine, Medical Faculty, University of Heidelberg, Heidelberg, Germany; 3 Institute for Psychosocial Prevention, Center for Psychosocial Medicine, Medical Faculty, University of Heidelberg, Heidelberg, Germany

**Keywords:** Personality functioning, Maladaptive traits, Personality disorder, Childhood trauma

## Abstract

**Purpose of Review:**

Childhood trauma is an important risk factor for the development of personality disorders (PDs), yet most research has been devoted to categorical models of personality pathology. Considering the introduction of a dimensional PD model with ICD-11, we review current findings related to various forms of childhood trauma, and PDs, operationalized in the form of personality functioning and maladaptive traits. We focus on the magnitude of associations and examine specific relationships between emotional and physical trauma with areas of personality functioning and single traits.

**Recent Findings:**

Two studies showed a strong association between childhood trauma and personality dysfunction. Seven studies, including clinical and forensic samples, demonstrated heterogeneous associations between various forms of childhood trauma and maladaptive traits. Overall, four studies indicated a slightly stronger association between personality dysfunction, maladaptive trait expression, and higher levels of emotional trauma than for physical or sexual trauma. Regarding specific trait domains and childhood trauma, most studies yielded the strongest associations for either psychoticism or detachment.

**Summary:**

Research on childhood trauma and dimensional PD models (i.e., personality functioning and traits) has the potential to contribute to a better understanding of their complex relationship. However, high intercorrelations among different types of childhood trauma, areas of personality functioning, and trait domains increase the difficulty of disentangling single effects. More research is needed including clinical and non-Western samples, especially considering the upcoming ICD-11 classification.

## Introduction

Traumatic experiences during childhood are associated with severe health consequences that can manifest at a young age and perpetuate long into adulthood [1–3]. These include an increased lifetime risk for various mental disorders [3]. The term “childhood trauma” is a collective definition for traumatic experiences in childhood, i.e., before the age of 18 years, which includes emotional and physical abuse, as well as neglect, and sexual abuse [4]. A severe and often long-lasting group of mental disorders associated with childhood trauma are personality disorders (PDs) [3].

Longitudinal studies showed that young adults with a history of abuse or neglect in childhood have a four-fold increased risk of developing a PD [4]. A recent meta-analysis by Porter and colleagues [5] and complementary analyses by Kleindienst et al. [6] suggest that individuals diagnosed with borderline personality disorder (BPD) are 1.62 times more likely to report childhood adversity than other psychiatric groups (including mood disorders, psychosis, and other PD types), with especially high effect sizes for emotional abuse and neglect [5].

Even though there is widespread consensus on a major etiological role for childhood trauma in the pathogenesis of PDs, with the majority of research devoted to BPD (see Gunderson et al. [7] for a review), developmental pathways and causality, as well as disorder-specific effects, remain debated [8–13]. Particularly the (1) magnitude and (2) specificity of observed effects between childhood trauma and PDs are heterogeneous [5]. Indeed, several studies reported associations of childhood abuse and neglect to various PD types and dimensional PD symptoms, including subclinical personality impairment [14–18]. However, personality impairment below the threshold of a PD is not captured in categorical PD classifications [14]. Moreover, there is general consensus that current PD diagnoses in DSM-IV, DSM-5 Section II, and ICD-10 lack reliability, validity, and clinical utility [19–24], possibly contributing to the heterogeneous results concerning their links with childhood trauma.

These deficits have led to the development of dimensional PD models, namely the DSM-5 Alternative Model for Personality Disorders (AMPD) and the upcoming ICD-11 PD chapter. Both define personality pathology along a continuum of personality functioning and maladaptive traits, rather than solely describing distinct types. Thus, this paradigm shift in PD classification represents an eligible opportunity to expand and clarify the understanding of magnitude and specificity concerning the association between various forms of childhood trauma and personality pathology [25••]

In this review, we therefore first introduce current conceptualizations of dimensional approaches to PD assessment in DSM-5 and ICD-11. Next, we summarize and evaluate currently available literature examining the relationship between various forms of childhood trauma, personality functioning, and maladaptive traits based on the AMPD and ICD-11. Finally, we discuss challenges and opportunities for future research.

## Dimensional Approaches to Personality Disorder Classification in DSM-5 and ICD-11

### The Alternative Model for Personality Disorders (AMPD)

The AMPD in DSM-5 Section III (“Emerging Measures and Models” [26]) incorporates two novel criteria for PD assessment: a severity continuum of impairment in personality functioning (Criterion A) and a hierarchical model of maladaptive personality traits (Criterion B). The formulation of Criterion A is based on the notion that impairments in self- and interpersonal functioning are at the core of personality pathology and thus inherent to all PD types [27, 28]. Along with this definition, an operationalization for dimensional severity assessment is provided in form of the Level of Personality Functioning Scale (LPFS [27]). The LPFS is a clinician-rating scale that distinguishes four elements of personality functioning (self: identity, self-direction; interpersonal: empathy, and intimacy) and 5 levels of impairment severity (0 = little or no impairment; 4 = extreme impairment) [26]. Criterion B provides five higher order trait domains (negative affectivity, detachment, antagonism, disinhibition, and psychoticism), designed to capture pathological personality characteristics [26]. The broad domains represent maladaptive extremes of the Five-Factor Model [23, 26] and are in turn subdivided into 25 empirically derived trait facets [29]. The DSM-5 Personality and Personality Disorders Workgroup also provides a corresponding self-report measure with the Personality Inventory for DSM-5 [29]. However, despite its dimensional diagnostic approach, the AMPD is a hybrid model and provides continuity with traditional PD classification: it retains six categorical DSM-IV PD diagnoses (antisocial, avoidant, borderline, narcissistic, obsessive-compulsive, and schizotypal), each complemented with descriptions along Criteria A and B [26].

### Personality Disorder in ICD-11

The ICD-11 nomenclature replaces the traditional PD types, which had remained virtually unchanged since their introduction in ICD-6 in 1948 [30]. Coming into effect on January 2022, the WHO classification adapts a dimensional system to personality pathology assessment on a single continuum of general PD severity [31]. In ICD-11, levels of personality functioning constitute the core of PD assessment: Personality pathology is characterized “by problems in functioning of aspects of the self (e.g., identity, self-worth, accuracy of self-view, self-direction), and/or interpersonal dysfunction (e.g., ability to develop and maintain close and mutually satisfying relationships, ability to understand others’ perspectives and to manage conflict in relationships)” […] [32]. The WHO taxonomy [32] distinguishes four levels of personality functioning: “personality difficulty,” “mild PD,” “moderate PD,” and “severe PD.” Moreover, PD symptoms may manifest within different areas of life (e.g., personal or work-related) and exhibit different qualities (e.g., emotional, cognitive, and behavioral). In addition to severity levels, ICD-11 provides five pathological trait domains (negative affectivity, detachment, disinhibition, dissociality, and anancastia) and a borderline pattern specifier that can be optionally assigned to describe prominent personality characteristics [31].

## Childhood Traumatic Experiences and Personality Functioning (Criterion A)

A literature research on childhood traumatization and personality functioning according to the AMPD and ICD-11 identified two studies, both from perspective of the AMPD [25••, 33••] (see Table 1 for an overview). Gander and colleagues [33••] investigated the mediating role of attachment on the relationship between childhood trauma and levels of personality functioning. Their sample consisted of 112 adolescents from a clinical inpatient setting and 87 adolescents from the general population. Childhood traumatization was defined and measured by Bernstein’s Childhood Trauma Questionnaire (CTQ) [35], a self-report questionnaire, which assesses emotional and physical abuse and neglect as well as sexual abuse. Personality functioning was measured with the Levels of Personality Functioning Questionnaire (LoPF-Q 12–18) for adolescents from 12 to 18 years [36]. Concerning the *magnitude* of the overall relationship between childhood trauma and personality dysfunction, this study demonstrated a strong correlation between the mean scores of the CTQ and LoPF-Q across the entire sample (*r* = .50) [33]. Examining the *specificity* of different traumatic effects, emotional trauma (including emotional abuse and neglect) was twice as strongly related to levels of personality dysfunction (*r* = .51) than physical traumata (including physical abuse, neglect, and sexual abuse; *r* = .25). Regarding the higher order domains of self- and interpersonal dysfunction, trauma was slightly more related to self-dysfunction (*r* = .48) than to interpersonal dysfunction (*r* = .40), although this trend for specificity is rather low.

**Table 1:** Overview of studies on childhood trauma, personality functioning, and/or maladaptive traits according to DSM-5 AMPD, identified by our literature search as published between 2017 and 05/2021

Title	Author	Year of publication	Measures of childhood trauma	Measures of personality pathology	*N* (total)	Main results
Unresolved attachment mediates the relationship between childhood trauma and impaired personality functioning in adolescence	Gander et al.	2020	Childhood Trauma Questionnaire (CTQ [34])	Levels of Personality Functioning Questionnaire (LoPF-Q 12-18 [36])	112 (clinical), 87 (community-based)	Childhood trauma and levels of personality dysfunction correlated strongly (*r* = .50). The associations between emotional abuse and neglect and the domains of identity, empathy, self-direction, and intimacy were mediated by the severity of attachment trauma.
Personality functioning, maladaptive traits, and childhood trauma	Back et al.	2020	Childhood Trauma Questionnaire (CTQ [35])	Levels of Personality Functioning Scale – Self Report (LPFS-SR [37]) Personality Inventory for the DSM-5 Brief Form (PID-5-BF [40])	473	Childhood trauma and levels of personality dysfunction and traits are strongly, albeit differentially associated to retrospective reports of childhood trauma.
Pathways to incarceration: an examination of childhood maltreatment and personality psychopathology in incarcerated adults	Boland et al.	2020	The Family Health History Questionnaire (FHHQ [1])	Personality Inventory for the DSM-5 Brief Form (PID-5-BF [40])	180 (forensic)	Childhood maltreatment variables are positively associated with maladaptive personality domains broadly (*r* = .18–31). Evidences were found for a mediation pathway from childhood maltreatment to maladaptive personality to adult criminal behavior.
PID-5 trait mediation of childhood maltreatment effects	Veith et al.	2017	The Violent Experiences Questionnaire [39•, 41, 42] Sexual abuse and assault self-report [43]	Personality Inventory for the DSM-5 (PID-5 [44])	526	Childhood physical abuse was associated with maladaptive traits (*r* = .12–.30) broadly. Four out of five maladaptive trait domains almost fully mediated the effects of childhood physical abuse on internalizing and externalizing symptoms.
Assessing the relationships between self-reports of childhood adverse experiences and DSM-5 alternative model of personality disorder traits and domains: A study on Italian community-dwelling adults	Borroni et al.	2019	Childhood Abuse and Trauma Scale (CATS [46])	Personality Inventory for the DSM-5 (PID-5 [44])	369	Childhood trauma was differentially, positively associated with maladaptive traits and the highest correlation was found with psychoticism.
Trauma-related dissociation is linked with maladaptive personality functioning	Granieri et al.	2018	The Traumatic Experiences Checklist (TEC [46])	Personality Inventory for the DSM-5 Brief Form-Adult (PID-5-BF [40])	322	Childhood trauma was differentially, positively associated with maladaptive traits and the highest correlation was found with psychoticism. Moreover, dissociation acted as a partial mediator between traumatic experiences and overall level of maladaptive traits.
Personality pathology among adults with history of childhood sexual abuse: Study of the Relevance of DSM-5 proposed traits and psychobiological features of temperament and character	Hemmati et al.	2020	Childhood Sexual Abuse (CSA [47])	Personality Inventory for the DSM-5 (PID-5 [44])	447 (43 victims of sexual abuse)	Victims of childhood sexual abuse demonstrated significantly higher levels of antagonism and psychoticism compared to adults without childhood sexual experiences.
The role of DSM-5 borderline personality symptomatology and traits in the link between childhood trauma and suicidal risk in psychiatric patients	Bach and Fjeldsted	2017	CTQ [35]	Personality Inventory for the DSM-5 (PID-5 [44])	124 (clinical)	Childhood trauma was associated with maladaptive personality subfacet traits (*r* = .09–.37). The authors observed the highest correlations of childhood traumatic experiences with two subfacets of psychoticism, namely suspiciousness and perceptual dysregulation. The two subfacets mediated the relationship between early trauma and subsequent suicidality in individuals with borderline personality disorder.

In the second study, Back and colleagues [25••] investigated the relationship between childhood trauma (CTQ [35]) and the Levels of Personality Functioning Scale–Self Report (LPFS-SR [37]) in a community-based sample of 437 young adults (18–30 years) analyzed with structural equation modeling. Interestingly, the *magnitude* of the overall association between childhood trauma and personality dysfunction was exactly as strong as reported by Gander and colleagues [33••] (*β* = .51). However, this study did not report a comparably obvious *specificity* of effects as in the study of Gander and colleagues [33••], although the path coefficient was slightly larger for personality dysfunction and emotional trauma (*β* = .56) than for physical trauma (*β* = .47).

Even though current research on childhood trauma and personality functioning according to DSM-5 and ICD-11 is sparse, both studies emphasize a strong relationship between childhood trauma and impairment in personality functioning. Interestingly, the two studies observed similar patterns in an adolescent, predominantly clinical sample, and a community sample of young adults. Taken together, both studies highlight a strong association of childhood maltreatment and personality pathology along a continuum including sub-threshold and milder forms of personality impairment [14]. Considering the specificity of traumatic effects, both studies indicate that past emotional trauma may be more relevant in the context of personality functioning than physical trauma. The fact that the sample of Gander and colleagues [33••] consisted predominantly of clinical individuals, with adolescents being less susceptible of the retrospective bias concerning their report of traumatic experiences than adults [34, 35], may enhance the validity of specific effects concerning emotional trauma. However, childhood trauma appeared to have a comparably strong relationship to all domains (e.g., self and other) of Criterion A, although a trend toward childhood trauma and dysfunction in the domain “self” was observed in the two studies [25••, 33••]. Further research including longitudinal designs are needed that require balanced clinical groups (including more categorical PD types) to ensure the validity of the observed relationships. Moreover, at the time of writing this review, studies on the new model in ICD-11 are still lacking, highlighting the relevance of further research on dimensional models.

## Childhood Traumatic Experiences and Maladaptive Traits (Criterion B)

In the tradition of the empirically derived and cross-culturally replicated Five-Factor Model of personality, the AMPD and ICD-11 both integrate maladaptive personality traits in the assessment of personality pathology [38]. While the assignment of personality domains is an optional feature in ICD-11, in the AMPD this step is mandatory and serves to integrate distinct PD types into a hybrid-dimensional-categorical model [26]. Consequently, Criterion B provides an integration of traditional PD diagnoses into an empirical framework of personality along the spectra of internalizing and externalizing behavior [23]. Applied to childhood trauma, the study of maladaptive personality traits may enhance our understanding of *magnitude* and especially *specificity* of effects of various early trauma types on divergent pathological trait variants.

Until now, seven studies examined the associations between early traumatic experiences in childhood and maladaptive personality traits. The studies included in this review are based on the AMPD; no study could be identified with respect to current ICD-11 trait measures (see Table 1 for an overview).

Veith and colleagues [39•] examined the effects of physical and sexual abuse on Criterion B, as well as the trait’s association to internalizing and externalizing symptoms in a sample of 526 adults. Four out of the five domains (antagonism, negative affectivity, detachment, and disinhibition) almost fully mediated the effects of childhood physical abuse on internalizing and externalizing symptoms. Internalizing symptoms were best predicted by negative affectivity and detachment, whereas externalizing symptoms were best predicted by disinhibition and antagonism [39•]. Concerning the magnitude and specificity of the traits’ associations with physical and sexual abuse in childhood, correlations ranged from small to medium (*r* = .12–.30). The highest effects were consistently found for sexual abuse across all five trait domains whereas the largest single effect was observed for childhood physical trauma on detachment.

Replicating the findings of Veith et al. [39•], Back et al. [25••] also reported detachment as highest correlate of childhood trauma. Detachment in the AMPD, characterized by social withdrawal and lack of affect as well as depressiveness and avoidance of closeness [23], exhibits a strong conceptual proximity to trauma-associated *avoidance* (characterized by dissociations, denial, or emotional “numbness”), depicting typical symptoms of post-traumatic stress disorder (PTSD) [48]. Therefore, the authors concluded that consequences of early traumatization may be reflected in a personality characterized by withdrawal, with proximity to symptoms as observed in PTSD [25••]. Extending the results of Veith et al. [39•], Back et al. [25••] additionally examined the effects of emotional abuse and neglect, reporting a high overall magnitude of correlations (*β* = .50) as well as higher total associations of Criterion B with *emotional* compared to physical trauma. Interestingly, they observed that especially emotional trauma is more highly associated with the internalizing trait spectrum (e.g., detachment and negative affectivity [39•]) than with externalizing personality traits (e.g., disinhibition and antagonism [39•]).

In two studies from Italy, Granieri and colleagues [49•] and Borroni and colleagues [50•] examined the relationship between traumatic experiences in childhood and maladaptive traits in adult community samples (*N* = 322 and *N* = 369). While Granieri [49•] screened childhood trauma by the Traumatic Experiences Checklist [46] measuring discrete traumatic events, Borroni and colleagues [50•] employed the Childhood Abuse and Trauma Scale, assessing subjective reports of emotional and physical abuse/neglect as well as sexual abuse [45]. Concerning the magnitude of association between childhood trauma and maladaptive traits, correlations in both studies range from small to medium (*r* = .12–.36). Matching the findings of Back et al. [25••], Borroni et al. [50•**]** found higher correlations for emotional abuse and neglect than for physical and sexual abuse/neglect. Both studies report the largest joint effects of childhood trauma on the trait psychoticism (describing odd, eccentric, or unusual behaviors, cognitions, and perceptual as well as cognitive impairments; [23]). Borroni et al. [50•] attributed this association to the shared variance between childhood trauma and dissociative tendencies as another well-documented trauma-reactive coping strategy. In favor of this conclusion, mediation analyses by Granieri et al. [49•] showed that dissociation acted as a partial mediator between traumatic experiences and overall level of maladaptive traits. These results match a large body of literature demonstrating a link between complex trauma and dissociative experiences [51]. Moreover, stress-related dissociation is a key symptom of BPD, linked to other core domains of PDs (see Krause-Utz [52] for a review). In line with the results of Veith et al. [39•] and Back et al. [25•], *psychoticism*, like *detachment*, shows conceptual overlaps with trauma-associated symptoms as observed in PTSD [48].

In distinction to population-based samples, some studies examined the association between childhood trauma and Criterion B using clinical, forensic, and high-trauma samples: Bach and Fjeldsted [53**••**] investigated a sample of 124 non-psychotic adult outpatients using the general score of the CTQ [35] as a measure of childhood trauma. The results demonstrated comparable patterns to the studies of the Italian colleagues [49**•**, 50**•**]. With a magnitude of trauma effects ranging from small to medium (*r* = .09–.37), Bach and Fjeldsted [53**••**] observed the highest correlations of childhood traumatic experiences with two subfacets of psychoticism, namely suspiciousness and perceptual dysregulation. Additionally, the two subfacets mediated the relationship between early trauma and subsequent suicidality. Thus, personality facets related to psychoticism, reflecting dissociative/psychotic-like experiences, may depict intrusive trauma, supporting the results of Granieri et al. [49**•**] and Borroni et al. [50**•**], as well as the conceptual proximity to PTSD symptoms along with detachment [25**••**, 39**•**].

A study by Boland et al. [54**•**] examined childhood maltreatment, maladaptive personality traits, and adult criminal behavior in a forensic sample of prison inmates (*n* = 180). Childhood trauma was measured with the Family Health History Questionnaire, assessing childhood sexual, physical, and psychological abuse [1, 55]. Regarding the magnitude of effects, Boland et al. [54**•**] reported small to medium correlations for childhood trauma and maladaptive traits (*r* = .18–31), with highest effects for emotional traumatization. Additionally, Boland et al. [54**•**] demonstrated a mediation pathway from childhood trauma to maladaptive personality to adult criminal behavior, where emotional traumatization again showed highest loadings. Replicating Back et al. [25**••**], Gander et al. [33**••**], and Borroni et al. [50**•**], emotional trauma seems to entail a specific importance regarding pathological traits, as compared to physical or sexual trauma. Negative affectivity, a key domain of BPD in the AMPD [26], showed the highest and most consistent association with childhood trauma [54**•**]. This finding is in line with a previous study that found more severe childhood maltreatment histories to be associated with greater levels of negative affectivity [55]. Moreover, it matches theoretical and empirical considerations of early emotional trauma on affective instability, such as the high comorbidities of anxiousness and depressiveness within personality impairment [56, 60]. However, within the mediation analysis, the trait antagonism showed the highest loading on the mediator personality pathology. As suggested by the authors, negative affectivity is thought to be associated with childhood maltreatment broadly, rather than with specific forms of childhood abuse and neglect [54**•**], while antagonism may play a major role when inspecting adult criminal behavior as outcome. This seems plausible given the facets of antagonism (“Emotional cold,” “Propensity to manipulation,” “Delicacy” [23]) and their conceptual proximity to symptoms of antisocial PD (“Indifference to or rationalizing of having hurt,” such as lying and manipulative behavior [26]), a disorder particularly common in forensic settings [57, 58]. Lastly, Hemmati et al. [59**•**] investigated maladaptive traits and sexual assault, comparing 43 adults with childhood experiences of sexual abuse to 404 adults without such experiences. Victims of childhood sexual abuse demonstrated significantly higher levels of antagonism and psychoticism. Again, these results add to previous findings concerning the importance of trauma-reactive dissociation as elicited by Bach and Fjeldsted [53**••**], Granieri et al. [49**•**], and Borroni et al. [50**•**].

To summarize, all seven studies demonstrated significant associations between childhood traumatization and adult maladaptive personality traits in various samples (community, clinical outpatient, and forensic settings). Concerning the magnitude of observed effects, there was a large variation ranging from small, medium, to high associations. This heterogeneity stresses the need for further research and meta-analyses to estimate reliable and valid effect sizes. Moreover, the variability of measures for childhood traumatization calls for employing more convergent instruments (including emotional trauma), as well as to inspect additional possible mediators explaining the variations in effect sizes.

Concerning the specificity of trauma effects, only three studies included measures for emotional abuse and or neglect, with all demonstrating higher associations to maladaptive trait domains than for physical and/or sexual abuse/neglect. This pattern matches empirical findings on Criterion A [25**••**, 33**••**] and BPD [5], underlining the importance of emotional traumatic experiences for various aspects of personality pathology (dimensional vs. categorical, trait- or severity-based).

Concerning specificity of maladaptive traits, four out of seven studies highlight the relative importance of psychoticism, linked to trauma-associated dissociation as a prominent post-traumatic and complex trauma coping mechanism. These studies therefore suggest that complex traumatic experiences in childhood might imprint on dissociative prone personality. As childhood trauma was related to various maladaptive traits, the studies support the notion of accumulating effects of abuse and neglect on personality. Regarding the higher order spectra of internalizing and externalizing trait domains, the reviewed studies hint towards a slightly higher effect for childhood trauma on domains of the internalizing spectrum (e.g., higher correlations with negative affectivity, detachment, and psychoticism).

## Conclusions as well as Possible Clinical and Scientific Implications

In this review, we summarized available research on associations between childhood traumatic experiences (i.e., emotional, physical, and sexual abuse and neglect) and dimensional models of personality pathology (i.e., personality functioning and maladaptive traits) according to DSM-5 Section III and ICD-11. We focused on the *magnitude* of the overall association as well as on the *specificity* of various traumatic effects on the domains of personality functioning (self- vs. interpersonal functioning) and spectra of maladaptive traits (internalizing and externalizing). Our review included eight studies, published between 2017 and 2021, that examined personality pathology as defined in the AMPD of the DSM-5 in the context of childhood trauma. To date, no study has been published on ICD-11 measures of personality pathology and childhood trauma.

Overall, all included studies reported significant associations between physical, emotional, and sexual trauma with personality functioning and/or maladaptive traits. The observed effects were mostly of comparable magnitude and add to the large body of research conducted on childhood trauma and personality pathology [5]. Two studies demonstrated an overall strong association of personality functioning and childhood trauma, while seven studies found a rather mixed pattern with regard to maladaptive traits (ranging from mild to strong associations).

Based on the findings of four studies assessing both physical and emotional trauma, we conclude that early emotional trauma may be of special importance regarding personality impairments. This highlights the relevance of theoretical considerations by Linehan [56, 60] such as Fonagy and Bateman [61] and is in line with recent meta-analytic findings on BPD [5]. According to Linehan [56, 60], BPD primarily entails emotion dysregulation, emerging from transactions between biological vulnerabilities and an emotionally invalidating developmental context. Likewise, emotional dysregulation mediates the relationship between borderline pattern symptoms and childhood emotional abuse [62]. Biological research on early trauma suggests that trauma exposure in childhood profoundly modifies the way in which emotional information is processed and prioritized [63]. Increased neurophysiological sensitivity to emotional stimuli, exaggerated neurophysiological responses to emotional stimuli, and absence of effective neurophysiological inhibitory control [63] (see Gunderson et al. [7] for a review) have equally been stated to biologically underlie borderline patterns [56, 60]. As the capacity for emotion regulation is mapped to the identity sub-domain in self-functioning of Criterion A [64], one could speculate that this may explain the slightly higher effect of emotional trauma to self-functioning observed by Gander and colleagues [33**••**]. According to Fonagy and Bateman, a lack of awareness to own emotional and cognitive states, resulting from absent emotional mirroring by early caregivers, in turn, is thought to hinder the development of own emotional and cognitive states as distinct to those of others. This lack of self-other distinction is an important antecedent of mentalizing deficits, associated with identity and interpersonal problems [61]. Therefore, we conclude that the relationship between emotional trauma and dimensional personality impairment importantly depicts and integrates theories of BPD, underlining their relevance in understanding PD.

Moreover, we found evidence for a specific pattern between trauma and the internalizing spectrum (negative affectivity, detachment, and psychoticism). In particular, the domains of psychoticism and detachment encompass trauma-specific (maladaptive) coping strategies such as dissociation and avoidance, which share strong conceptual and empirical overlap with PTSD [65]. We therefore conclude that childhood trauma may imprint on personality traits eventually reflecting trauma-reactive, long-term internalizing coping strategies.

Although theoretical narratives support the specificity of emotional trauma and the internalizing spectrum [38], empirically the domains of childhood trauma, personality functioning, and maladaptive traits mostly demonstrate moderate to high intercorrelations [25**••**, 39**•**, 66]. Thus, it remains difficult to disentangle single effects between elements of personality functioning, trait domains, and types of childhood trauma (emotional, physical, and sexual). However, this may not only reflect the complex etiopathogenesis of personality pathology but may also be owed to the methods and instruments. Disentangling individual domain effects is difficult because the areas of personality functioning exhibit substantial intercorrelations (mean correlation of ≥.8 between the LPFS-SR subscales) [37]. Most importantly, however, is the conclusion that from the perspective of both severity and traits, any type of childhood maltreatment (emotional, physical, or sexual) has complex, cumulative, and likely long-lasting associations [7] with personality pathology along a continuum (even in individuals who do not meet the categorical diagnosis of a PD).

A number of opportunities and challenges for research and clinical practice arise from these findings: (a) identifying specific psychosocial pathways to personality pathology remains a challenge even when considering severity and maladaptive traits. Nevertheless, dimensional PD models may enhance possibilities of early prevention and treatment. Employing a dimensional framework not only allows for a more fine-grained detection of milder forms of impairments at earlier stage, but also the assignment of appropriate intervention strategies. In the light of the major changes in PD classification with ICD-11, it will be crucial to conduct research on levels of personality functioning and early traumatic experiences. This is particularly significant as current literature on childhood trauma has been predominantly devoted to maladaptive traits and there has not been a single study on the ICD-11 model to date. Moreover, despite their similarities, AMPD and ICD-11 have notable differences such as the featured maladaptive traits with ICD-11 integrating a domain of anancastia instead of psychoticism. Thus, studies of PD severity and style from a joint perspective of the AMPD and ICD-11 are needed, which is fostered by the development of DSM- and ICD-compatible instruments [67]. (b) The large variability in measures and definitions of childhood trauma makes it difficult to merge effects reliably and validly. Our review shows that it is crucial to choose instruments of childhood trauma, which include emotional maltreatment, when inspecting future associations to personality pathology. Moreover, the studies listed here all employed self-report instruments that carry well-documented limitations (e.g., recall bias [68]). (c) As most studies rely on retrospective approaches, it will be important to track the relationship between childhood trauma and personality pathology through longitudinal studies as well as meta-analysis. Employing dimensional measures of personality pathology may provide the opportunity to capture dynamic changes in personality impairment and traits, and thus in a more valid and reliable fashion than by means of categorical models. (d) The studies reviewed here included predominantly Western samples. However, personality functioning and traits must be considered in the context of cultural aspects such as social norms and values [69]. Thus, further research is needed to examine the generalizability of the findings presented here across cultural differences. (e) Lastly, conceptualizing personality impairment along a dimension provides an important opportunity towards reducing the stigma surrounding personality pathology [70], especially in the case of BPD [71, 72].

## Data Availability

Not applicable.
